# BRCA1 A-Complex fine tunes repair functions of BRCA1

**DOI:** 10.18632/aging.100334

**Published:** 2011-05-30

**Authors:** Janelle L Harris, Kum Kum Khanna

**Affiliations:** ^1^ Queensland Institute of Medical Research, Signal Transduction Laboratory, Brisbane, Queensland 4029, Australia

Germline mutations in BRCA1 increase the risk of breast and ovarian cancer, but the specific pathways driving breast and ovarian cancer development in BRCA1 mutants are currently unclear [1]. Several studies have demonstrated that BRCA1 is required for cellular responses to DNA double strand breaks (DSBs) and homologous recombination (HR), although its exact role in these processes is unclear. BRCA1 contains an N-terminal RING domain and two C-terminal BRCT repeats. The BRCA1 RING domain imparts ubiquitin ligase activity to BRCA1 through interaction with its key binding partner BARD1 [2]. BRCT repeats contribute to transcriptional and DNA repair function of BRCA1, and cancer associated mutations that disrupt BRCT motif impair these activities [3].

BRCA1 forms at least 3 distinct complexes (BRCA1 A, BRCA1 B and BRCA1 C) through association of different adaptor proteins with BRCT motif. A-Complex function has been the subject of considerable research interest in the last several years, and is the focus of a study by Dever *et al*. in this issue of Aging [4] as well as other recent studies [5, 6]. The A-Complex consists of BRCA1 in association with the ubiquitin interacting motif (UIM) containing protein RAP80, the deubiquitinylating (DUB) enzymes BRCC36 and BRCC45, MERIT 40, and the adaptor protein Abraxas [7-10]. The A-Complex is thought to target BRCA1 to ionising radiation (IR) inducible foci through interaction of RAP80 with K63 poly-ubiquitin chains at sites of DSBs, in particular on H2AX [7, 11-13]. This RAP80 recruiting ubiquitinylation is performed by the E2 ubiquitin conjugase Ubc13 and the E3 ligase RNF8, which are targeted to breaks by MDC1 [14-16]. The critical role of ubiquitinylation as a recruitment signal at DNA damage sites is highlighted by the hypersensitivity of Ubc13 and RNF8-deficient cells to irradiation [14-17].

The new study by Dever *et al*. indicates that disruption of the BRCA1 complexes through mutation in BRCT motif causes genomic instability due to an increase in recombination [4]. Specifically, the authors explored the function of the BRCA1 BRCT mutant K1702M, in which BRCT mediated phosphoprotein interactions are disrupted. They found that expression of K1702M caused radiosensitivity compared to wild-type complemented BRCA1 deficient cells. Intra-chromosomal recombination is commonly assessed using a flow cytometry based DR-GFP recombination assay. Dever *et al*. used this method to determine that expression of the K1702M mutant was associated with elevated recombination compared to wild-type BRCA1, indicating that the K1702M mutant disrupts critical negative regulation of recombination [4]. It was unclear if this increased recombinational activity was due to the accumulation of cells in S/G2 [4]. The hyper-recombination of K1702M cells is in line with two other studies which found that transient depletion of non-BRCA1 members of the A-Complex caused an increase in recombination, using a similar DR-GFP approach [5, 6]. Interestingly, these studies found that siRNA depletion of BRCC36, one of the A-Complex deubiquitinylating enzymes, increased recombination activity to the same extent as RAP80 depletion[5, 6], seemingly contradicting earlier studies that reported reduced HR by depletion of the components of A-Complex [13, 18] and the reason for this lack of concordance is unclear. In support of the role of the BRCA1 BRCT domain and the A-Complex in negative regulation of recombination [4-6], is the finding that a balance between histone ubiquitinylation by RNF8/ Ubc13 and deubiquitinylation by BRCC36/RAP80 is critical for maintenance of genomic stability [19]. This supports a model where recruitment and subsequent deubiquitinylation activity of the A-Complex terminates DNA break associated ubiquitinylation. The importance of balanced poly-ubiquitin synthesis and removal at the break site is highlighted by regulation of FANCD2 [20]. Failure to ubiquitinate or deubiquitinate FANCD2 confers sensitivity to cross-linking agents and impairs recombination activity.

Recent work by Hu *et al*. showed that the hyper-recombination activity in RAP80 deficient cells is dependent on BRCA1, since double depletion of RAP80 and BRCA1 suppressed the increased HR observed in RAP80 deficient cells [6]. Dever *et al*. extended on this by showing that the hyper-recombination observed in BRCT-mutant expressing cells is dependent on BRCA1 ubiquitin ligase activity [4]. A BRCA1 RING domain mutation known to block BRCA1 ubiquitin ligase activity (I26A) was able to diminish increased recombination observed with the expression of the BRCT mutant K1702M [4]. Given the accumulation of K1702M mutant expressing cells in S/G2 phase of the cell cycle reported in this study [4], it would be interesting to know what effect the I26A/ K1702M double mutant has on cell cycle. Taken along with the hyper-recombination of BRCC36 depleted cells, these results support a model where the A-Complex negatively regulates the pro-recombination activity of an ubiquitinylation-dependant BRCA1 pathway.

**Figure 1.**
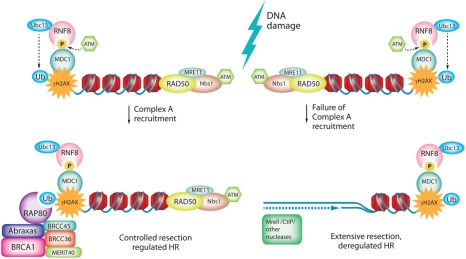
A-Complex regulates DSB resection

Some questions raised from this conclusion are: What recombination mechanism is the A-Complex regulating? What effects does A-Complex disruption have on the structure of chromatin surrounding a break site? How does A-Complex recruitment and deubiquitinylase activity affect recruitment of other factors? The mechanism of hyper-recombination in A-Complex disrupted cells has been addressed by Hu *et al*. [6] and in the Dever *et al*. [4] study. They found that ablation of the BRCA1-Abraxas interaction promotes extensive resection, as visualized by an increase in single stranded DNA abundance and chromatin retention of RPA and RAD51[4, 6]. Elevated binding of RPA and RAD51 is indicative of more abundant or longer stretches of resected single stranded DNA. In fact Hu *et al*. demonstrated that a single I-SceI induced DSB is resected further in cells lacking RAP80, by mapping RPA and Rad51 binding around the break by chromatin-immunoprecipitation (ChIP)-PCR [6]. Dever *et al*. directly measured single stranded DNA by detecting BrdU incorporation in non-denaturing conditions [4]. Both studies showed that disruption of BRCT interactions causes an increase in the abundance of single stranded DNA [4, 6]. Thus the hyper-recombination of A-Complex disrupted cells observed in several studies is the result of excessive resection, possibly due to loss of A-Complex catalysed termination of ubiquitinylation (deubiquitinylation) at breaks [19] (Figure 1).

Taken together these recent studies provide firm evidence that the BRCA1 A-Complex fine-tunes the repair function of BRCA1 by repressing excessive resection in a BRCT dependent manner. Interesting areas for further study may include elucidating the role of post-translational modifications in A-Complex formation and regulation. It would also be interesting to examine the interaction profile of BRCT mutants to identify mediators of excessive recombination. The hyper-recombination observed in BRCC36 depleted cells and the decreased recombination observed in Usp1 (FANCD2 deubiquitinylase) deleted cells highlight a central role for different ubiquitin marks on chromatin and repair proteins. Identification of A-Complex deubiquitinylase targets and downstream effects would provide valuable insight into the mechanism of hyper-recombination in A-Complex defective cells.

Double strand breaks are detected by the sensor protein complex Mre11-Rad50-Nbs1 (MRN), which recruits and activates the kinase ATM. Activated ATM phosphorylates many DNA repair proteins including MDC1. ATM phosphorylated MDC1 recruits the RNF8-Ubc13 E3 ubiquitin ligase which synthesizes K63 linked polyubiquitin on histones H2A and H2AX. RAP80 targets the A-Complex to breaks via the interactions of its ubiquitin interacting motifs (UIMs) with K63 linked polyubiquitin. Ubiquitin-dependent A-Complex localization to chromatin and subsequent deubiquitinylation fine tunes resection, resulting in tightly controlled recombination. Failure of A-Complex recruitment due to mutation in the BRCT motif, or depletion of A-Complex components including BRCC36/45 (deubiquitinylases) would alter the chromatin ubiquitinylation pattern. Such persistence or aberration of chromatin marks would change the complement and activity of repair proteins at the break site, resulting in de-regulation of repair. We propose that deubiquitinylase activity of the A-Complex restricts the recruitment and activity of nucleases, so disruption of the A-Complex leads to extensive resection and uncontrolled recombination.
